# Supplementary material for: Content-rich biological network constructed by mining PubMed abstracts
Source: BMC Bioinformatics. 2004 Oct 8;5:147. doi: 10.1186/1471-2105-5-147 (PMC528731; doi:10.1186/1471-2105-5-147)
Supplement: Additional File 2 — The original results of the above study (non-essential files are deleted to keep the file size under the limit set by BMC bioinformatics). [file 1471-2105-5-147-S2.bz2 › chilibotAdditionalFile2/dip10/592ID9262406E10965/html/ARHGAP1_CDC42.html]

 


 **ARHGAP1** and **CDC42** 
  
Found 46 abstracts in PubMed, retrieved 10.  
 

 What does Google say? 
 PDF only 
| .edu only 

---

**Interactive relationship** (e.g. stimulation, inhibition, etc)

**Stimulatory relationship**- The RhoGAP  [ **ARHGAP1** ]  domain of Gmip stimulates in vitro the GTPase activity of RhoA, but is inactive towards other Rho family proteins such as Rac1 and  **Cdc42** .  Ref: 12093360 Biochem J, 2002
**Neutral relationship**- Together, these findings indicate that during cytokinesis, MgcRacGAP, previously known as a GAP for Rac  **Cdc42** , is functionally converted to a RhoGAP  [ **ARHGAP1** ]  through phosphorylation by Aurora B.  Ref: 12689593 Dev Cell, 2003
- Proteins containing a RhoGAP  [ **ARHGAP1** ]  Rho GTPase activating protein domain usually function to catalyze the hydrolysis of GTP that is bound to Rho, Rac and or  **Cdc42** , inactivating these regulators of the actin cytoskeleton.  Ref: 12297274 FEBS Lett, 2002

**Non-interactive relationship** (e.g. studied together, co-existance, homology, etc.)

- The cycling of  **Cdc42**  between its on GTP bound and off GDP bound states is essential for its stimulation of cell growth and transformation, with an important aspect of this cycle being the regulation of the GTP hydrolytic activity of  **Cdc42**  by its GTPase activating protein Cdc42GAP  [ **ARHGAP1** ] .  Ref: 12501193 Biochemistry, 2002
- Ab initio methods were used to shed light on fundamental aspects of the enzymatic mechanism of guanosine triphosphate hydrolysis in the  **Cdc42**  Cdc42GAP  [ **ARHGAP1** ]  complex.  Ref: 11929266 J Am Chem Soc, 2002
- In the study presented here, we have tested this idea by examining three residues within the Cdc42GAP  [ **ARHGAP1** ] , which are missing in the GAP homology domain of the 85 kDa regulatory subunit p85 of the PI 3 kinase and are involved in specific interactions with switch domain residues of  **Cdc42** .  Ref: 12501193 Biochemistry, 2002
- While ExoT RhoGAP  [ **ARHGAP1** ]  stimulates actin reorganization through the inactivation of Rho, Rac and  **Cdc42** , the function of the ADP ribosylation domain is unknown.  Ref: 12807879 J Biol Chem< MedlineTA>J Biol Chem, 2002
- On the basis of the structural determinations of the  **Cdc42**  Cdc42GAP  [ **ARHGAP1** ]  complex, as well as the Ras RasGAP complex, it has been proposed that an arginine residue provided by the GAP called the arginine finger stabilizes charges developing on the guanine nucleotide during the transition state for GTP hydrolysis and is an important contributor to GAP stimulated catalysis.  Ref: 12501193 Biochemistry, 2002
